# Strengthening equity and anti-racism in women’s care: a quality improvement initiative reducing institutional maternal mortality in Brazil

**DOI:** 10.1186/s12939-025-02452-z

**Published:** 2025-04-23

**Authors:** Santiago Nariño, Jussara Francisca de Assis dos Santos, Tayna Brito, Livia Sanches Pedrilio, Paulo Borem, Claudia Garcia de Barros, Sebastian Vernal

**Affiliations:** 1 Northeastern University, Boston, MA USA; 2 Universidade Federal Fluminense, Rio de Janeiro, RJ Brazil; 3 Institute for Healthcare Improvement, Boston, MA USA; 4 Escritório de Excelência, Hospital Israelita Albert Einstein, São Paulo, SP Brazil

**Keywords:** Gender equity, Improvement science, Maternal death, Maternal health, Maternal mortality, Racism, Social justice, Obstetric care, Quality improvement

## Abstract

**Background:**

Circumstances that lead to maternal death are complex and multifactorial, including inequity and racism issues. Quality improvement (QI) strategies have demonstrated success in improving maternal outcomes. The Collaborative* Abraço de Mãe* (CAM) reduced the institutional maternal mortality rate (iMMR) by 34.2% from the baseline rate in 19 Brazilian maternity hospitals.

**Objective:**

To present the integration of anti-racism and equity strategies implemented during the CAM.

**Methods:**

A QI report assessing the CAM focused on strengthening the awareness of obstetric teams about ethnic-racial inequalities and institutional racism as social determinants of maternal outcomes. A mixed methods approach was used to understand the overall impact of the intervention. Measures included the *Anti-Racist Leadership Survey* and interviews (individual and grouped). Qualitative and quantitative data were applied simultaneously but independently, followed by a triangulated comparison to define convergences.

**Results:**

The domain with the highest average score was emotional resources and communication; the lowest was fundamental knowledge and translation of the knowledge in action. Interviews evidenced three categories: (A) equity and anti-racism training contributed to a more profound recognition of race and racism awareness, leading to a change in culture; (B) Priority change ideas and actions focusing on anti-racism and equity were demonstrated in several ways among the leaders; and (C) Challenges when equity is centralised in the care model. Triangulations revealed two convergences: (i) Evidence of a better understanding of ethnic-racial inequalities, institutional racism, and racism recognition by the leaders and participating institutions, and (ii) Resistance when trying to bring new content to the clinical staff, as well as a lack of tools when dealing with the emotional resources needed to confront interpersonal racism.

**Conclusion:**

With a significant reduction in iMMR, the CAM reveals that a QI intervention addressing inequities and racism issues is a feasible and promising approach to improve maternal outcomes within an equity-oriented model of care.

**Supplementary Information:**

The online version contains supplementary material available at 10.1186/s12939-025-02452-z.

## Significance

**What is already known on this topic?**

• Maternal deaths have decreased worldwide, yet pregnancy-related mortality continues to be a major public health concern, particularly in low- and lower-middle-income countries. The disparities in maternal deaths are shaped by factors beyond biological differences, including systemic issues such as inequity and racism.

**What does this study add?**

• The quality improvement Collaborative* Abraço de Mãe* (CAM) achieved a 34.2% reduction in the institutional maternal mortality rate across 19 participating maternity hospitals.

• This study demonstrates how CAM addressed ethnic-racial inequalities and institutional racism as key social determinants of maternal outcomes, incorporating equity and anti-racism training for obstetric staff as a vital component of its strategy.

**How might this study affect research and practice?**

• This study advocates for integrating equity and anti-racism training within healthcare systems to enhance the quality of care for all women.

• Our methodologies and findings may impact future research, clinical practice, and policymaking in maternal healthcare, especially in contexts affected by racial and socio-economic disparities.

**Supplementary Information:**

The online version contains supplementary material available at 10.1186/s12939-025-02452-z.

## Background

Although the Maternal Mortality Rate (MMR) has decreased globally in recent decades, pregnancy-related mortality remains a significant public health issue [1]. Moreover, considerable variations persist between and within countries, primarily related to income levels and resource distribution; currently, most women’s deaths occur in low- and lower-middle-income settings [1–3].

Circumstances leading to maternal death are complex and multifactorial, involving issues of inequity and racism [1, 4]. For instance, the United States has shown inequalities in MMR, particularly evident between white and black women and between non-Hispanic and Hispanic white women [5, 6]. Social and structural determinants, rather than biological factors, contribute to these ethnic disparities [1, 4–6].

In this global context, Brazil is not an exception; by 2018, more than 54% of all maternal deaths occurred among black women, who are twice as likely to die during pregnancy and childbirth compared to white women [7–10]. Regrettably, during the pandemic, these inequities became even more evident. Black women accounted for 1,095 of the pregnant and postpartum women who died from COVID-19, representing 54% of this group by March 2022. Moreover, pregnant and postpartum black women were more heavily impacted by SARS-CoV-2 than white women, totalling 5,941 cases in 2021, which also resulted in a higher number of hospitalisations and use of intensive care beds [11].

In the pursuit of health equity during pregnancy and postpartum, quality improvement (QI) initiatives have been implemented to promote safe care and enhance maternal outcomes [12–14]. In addressing inequities and racism, a multidisciplinary working group from the *National Partnership for Maternal Safety* developed a bundle aimed at reducing peripartum disparities. The objective is to inform health professionals about racial and ethnic inequities that directly influence pregnancy-related outcomes. This approach targets race conditions that can be modified within a healthcare system to ensure equitable and safe assistance for all women [15]. 

In front of this, the *Hospital Israelita Albert Einstein* (HIAE), along with the Institute for Healthcare Improvement (IHI), conducted a Breakthrough Series Collaborative (BTS) called Collaborative* Abraço de Mãe* (CAM), aimed at reducing the institutional MMR (iMMR) in Brazilian public maternity hospitals [16]. From November 2019 to March 2021, this initiative decreased the iMMR baseline by 34.2% (from 83.7 to 55.0 deaths per 100,000 live births) across 19 participating maternal care units. In addition to QI approaches to reduce maternal deaths, this Collaborative also concentrated on enhancing obstetric teams’ awareness of ethnic-racial inequalities and institutional racism as social determinants of maternal outcomes while creating tangible actions to address inequities within the system. This QI report aimed to outline the equity and anti-racism training for obstetric staff from 19 participating institutions, emphasising it as a critical complementary component of the objectives achieved by the CAM.

## Methods

### Context

In 2010, the Brazilian Ministry of Health introduced the *National Policy for Integral Health of the Black Population* to address systemic racism in the unified public health system [17]. This policy encourages strategies to adapt healthcare facilities for the Black population with a strong focus on data stratification by race/colour and ethnicity to document disparities and illustrate race-conscious healthcare programs [17, 18]. Nevertheless, this is not easy; transforming this plan into daily care action remains challenging for stakeholders [19]. Hospitals face numerous priorities and demands from national and state governments and, therefore, must choose to act on defined policy priorities rather than policy changes. A lack of understanding of structural racism at interpersonal and institutional levels may also contribute to the delay in implementing this strategy [19, 20]. Moreover, decisions in the Brazilian healthcare system are often made by experts (a paternalism pattern) rather than co-creating with people with lived experience or leaders from underrepresented populations, which may also impact the prioritisation of this public health plan [21]. In addition to the clinical-assistance QI activities aimed at reducing maternal deaths, the CAM also developed strategies aligned with the *National Policy for Integral Health of the Black Population.*

### Theory of change

The BTS methodology was selected for the CAM activities [22]. Two essential tools illustrate the theory of change in large-scale collaborations: the logic model and the driver diagram (DD).

The logic model visually represents the theory of change, detailing the actions needed to achieve short-, medium-, and long-term goals (Supplementary Table 1S). These actions involve QI activities aimed at reducing inequities in maternal deaths through interventions focused on equity and anti-racism.

The DD (as previously referred [16]) illustrates the theory of change within each participating institution by outlining the key ideas and concepts for promoting awareness of ethnic-racial inequalities while recognising racism as a social determinant of health. Although measuring racism poses challenges, this theory suggests it can be addressed by collaborating with obstetric teams to enhance awareness and integrate this new knowledge into daily practice through actionable change ideas. We hypothesised that focusing on raising awareness and improving education about ethnic-racial inequalities–along with developing change ideas through improvement science–would further reduce iMMR in the participating maternal care units.

### Study design

This study is a QI report assessing the CAM’s implementation from November 2019 to March 2021, focusing on equity and anti-racism interventions. The previous report [16] provides further information on QI interventions and CAM outcomes. As recommended, the Standards for Quality Improvement Reporting Excellence (SQUIRE 2.0) were followed [23].

### Participants

As previously reported [16], 19 public maternity hospitals were included. The leader was defined as the primary contact selected by the obstetric team, who must be at least 18 years old. There were no additional exclusion criteria. Contracted social workers from each service were also invited to participate voluntarily.

### Intervention

Following the BTS model, the equity and anti-racism training program was directed toward the leaders and social services professionals of each institution. The overarching aim was to provide equitable access to integrated, evidence-based care, helping the participating professionals raise awareness and expand their knowledge of equity and anti-racism issues. The HIAE and the IHI support team developed the intervention framework, conducting preliminary work and recruiting the QI teams to provide leadership, coordination, and in-practice coaching following the DD. Four Learning Sessions, totalling 16 h of training, were implemented during the intervention period for 19 leaders (one from each participating institution) and eight social workers.

### Study of the intervention

A mixed-methods approach was employed to grasp the overall impact of the intervention. Initially, qualitative and quantitative data were utilised simultaneously yet independently. Following data collection, the QI team analysed the two sets separately before conducting a triangulated comparison to identify convergences.

### Measures

- *Questionnaire*s. Pre- and post-test surveys were applied to the leaders who completed the equity and anti-racism training from the 19 participating institutions. The *Anti-Racist Leadership Survey* [24] (Supplementary Table 2S) uses the *Likert scale* to evaluate 42 items divided into five domains of anti-racist leadership: (I) fundamental knowledge, (II) race and racism awareness, (III) motivation and prioritisation, (IV) translation of the knowledge in action, and (V) emotional resources and communication. The survey was adapted to the Brazilian context and tested before use to ensure trans-cultural adaptability. Additionally, leaders’ demographic and maternity units' data were collected to assess variations across individuals and institutions. Survey responses enabled leaders to understand the areas they needed to focus on in their equity action plans. The pre-test survey was applied in October 2020, and the post-test was performed in June 2021.
- *Interviews*. Individual and group interviews were conducted to assess potential changes in the participating institutions, evaluate the knowledge acquired by the professionals involved, and analyse whether the training strengthened emotional and communication resources and their ability to apply the new learning in daily assistance. Two group interviews were organised: one with social workers participating in the equity and anti-racism training and the other with obstetric frontline staff. Data collection for the qualitative, interpretative, and exploratory analysis followed an inductive approach, utilising semi-structured interview techniques and virtual focus group interviews. The interview scripts were based on the five domains of the * Anti-Racist Leadership Survey* and the opportunities and challenges faced by obstetric teams in developing a service model centered on equity. Participants were invited via institutional email, with reminders sent through *WhatsApp*, WhatsApp Messenger (USA).

### Analysis

Data from pre- and post-test surveys were compiled separately. First, exploratory data analysis was conducted to assess general patterns and identify opportunities for improvement. An individual pre- and post-analysis was performed based on the sum of the scores for each of the five domains to understand each leader’s progress and pinpoint areas where they needed to focus their efforts. *Cronbach’s alpha coefficient* was calculated for each domain. *The Wilcoxon signed-rank* test was applied to compare scores for participants who completed both pre- and post-test surveys. Average ranges, standard deviations, and *P-values* are presented in tables as appropriate. Subgroup analysis was conducted based on demographic data. The significance level was set to α = 5%. Statistical analysis was performed using *SPSS*, v26 (USA).

Interviews were conducted using *Microsoft Teams*, Microsoft (USA), recorded, transcribed in *Microsoft Word*, Microsoft (USA), analysed, and coded. Coding began when the analysis units–quotes– were identified in the transcribed texts, omitting non-relevant or unrelated information. Data analysis followed the approach of open and axial coding: (i) Open Coding: synthesis, grouping, and relationship of the unit, facilitating the identification of patterns and the emergence of categories; and (ii) Axial Coding: abstraction, interpretation, and higher-level relationships among the experiences, senses, and meanings of those who participated in the interviews. The QI team carried out the coding and categorisation process. Quotes (paragraphs, sentences, or words) were summarised in tables. Coding was performed using NVivo *18*, v1.0 (USA).

We employed a structured triangulation process to ensure a comprehensive and rigorous cross-analysis of qualitative and quantitative data. First, we independently analysed the qualitative and quantitative datasets to identify emerging patterns and themes. Quantitative data from the *Anti-Racist Leadership Survey* were analysed, generating domain scores and subgroup analyses. Simultaneously, we coded qualitative interview transcripts, following an inductive approach. The final step involved a triangulated comparison, mapping thematic findings from the interviews against survey response trends to identify areas of convergence and divergence. This integration process provided a deeper understanding of how participants’ self-reported awareness and knowledge aligned with their qualitative reflections, enhancing the validity of our findings. Additionally, expert reviewers within the research team conducted independent checks to ensure the consistency and reliability of the cross-analysis.

## Results

### Surveys

Three leaders responded to the pre-test survey. All other pre-test survey respondents were social workers, not included in further analysis. Sixteen out of 19 leaders responded to the post-test survey: 75% were self-considered whites, 13% black, and 19% mixed-race (from the Portuguese “*Pardo*,” a mix between black and white). All of them were female, with a median age of 40.6 years old. Regarding their professional background, 15% were physicians, 85% were nurses, and 67% had more than ten years of work experience, with an average of 15.5 years in their professions. Considering the institutions from the 16 leaders, the number of beds varied from 30 to 500 wards, staff from 15 to 600 healthcare professionals, and women assistance from 100 to 5,000 monthly patients, where 30–85% were black. Table 1 summarises the post-test survey results and each domain’s reliability. The only domain with questionable internal consistency (*alpha coefficient =* 0.616) was motivation and prioritisation; all the rest were acceptable or good. The domains with more significant opportunities for improvement were Fundamental knowledge and translation of knowledge in action (average score of 3.53 for both).

**Table 1:** Post-test survey results by domains from 16 participating leaders

Domain	Average (Interval)	Minimum/Maximum	Cronbach’s alpha coefficient	Internal consistently
Fundamental knowledge	3.53 (1.33)	3.00/4.33	0.781	Acceptable
Race and Racism awareness	4.04 (1.22)	3.44/4.67	0.774	Acceptable
Motivation and prioritisation	3.77 (1.43)	3.14/4.57	0.616	Questionable
Translation of the knowledge in action	3.53 (1.78)	2.67/4.44	0.807	Good
Emotional resources and communication	4.10 (1.70)	3.20/4.90	0.806	Good

The average score for each item is presented in Table 2. None of them scored less than 3.1 or more than 4.4. The lowest score was in the translation of knowledge in the action domain, item 1 (3.13 ± 1.22). The highest score was in the Fundamental Knowledge domain, item 2 (4.31 ± 0.60), which discussed how the participants understand the Brazilian legacy in relation to black and mixed-race culture and its connection to the inequitable results from all institutions today.

**Table 2:** Post-test survey results by items from 16 participating leaders

Item	Domains– Average Score (Standard deviation) [Minimum/Maximum]				
	Fundamental knowledge	Race and Racism awareness	Motivation and prioritisation	Translation of the knowledge in action	Emotional resources and communication
1	4.25 (± 0.57) [3/5]	4.00 (± 0.73) [3/5]	3.87 (± 1.08) [1/5]	3.19 (± 1.22) [1/5]	4.13 (± 0.61) [3/5]
2	4.31 (± 0.60) [3/5]	4.13 (± 0.61) [3/5]	3.87 (± 0.61) [3/5]	3.50 (± 1.15) [1/5]	4.19 (± 0.75) [2/5]
3	4.00 (± 0.73) [3/5]	3.94 (± 0.85) [3/5]	3.87 (± 0.71) [3/5]	3.81 (± 0.75) [2/5]	4.25 (± 0.68) [3/5]
4	3.87 (± 0.61) [3/5]	4.25 (± 0.44) [2/5]	3.69 (± 0.70) [2/5]	3.69 (± 0.79) [2/5]	4.25 (± 0.68) [3/5]
5	3.88 (± 0.88) [2/5]	4.06 (± 0.68) [2/5]	3.75 (± 0.68) [3/5]	3.50 (± 0.63) [2/4]	4.00 (± 0.81) [3/5]
6	3.63 (± 1.02) [3/5]	4.19 (± 0.65) [3/5]	3.25 (± 1.00) [1/5]	3.25 (± 0.77) [2/5]	3.94 (± 0.77) [2/5]
7	3.94 (± 0.77) [3/5]	4.06 (± 0.77) [2/5]	4.13 (± 0.71) [2/5]	3.50 (± 0.81) [2/5]	4.13 (± 0.61) [3/5]
8	4.06 (± 1.06) [1/5]	4.00 (± 0.73) [3/5]	3.87 (± 0.80) [3/5]	-	3.75 (± 1.34) [1/5]
9	-	-	-	-	4.25 (± 0.68) [3/5]
10	-	-	-	-	3.50 (± 0.89) [2/5]

Subgroup analysis by leaders’ ethnicity and profession did not show significant differences in the average scores of each domain (data not shown).

As only three leaders responded, Table 3 illustrates the differences among these participants in their pre- and post-test results.

**Table 3:** Pre and post-test survey results by items from three participating leaders

Leader	Domains: Pre-test | Post-test (variation)				
	Fundamental knowledge	Race and Racism awareness	Motivation and prioritisation	Translation of the knowledge in action	Emotional resources and communication
1	3.75 | 3.25 (-0.50)	4.40 | 4.00 (-0.40)	3.57 | 4.00 (0.43)	2.89 | 3.44 (0.55)	4.20 | 4.10 (-0.10)
2	4.38 | 4.13 (-0.25)	3.90 | 4.67 (0.77)	3.71 | 4.29 (0.58)	3.89 | 3.89 (0.00)	3.90 | 4.00 (0.10)
3	3.38 | 3.63 (0.25)	4.50 | 4.56 (0.06)	2.86 | 3.86 (1.00)	2.11 | 2.67 (0.56)	4.40 | 4.40 (0.00)
**Average**	3.83 | 3.88 (0.05)	4.26 | 4.41 (0.15)	3.38 | 4.05 (0.67)	2.96 | 3.33 (0.37)	4.17 | 4.17 (0.00)

### Interviews

Individual interviews were conducted with 16 leaders of each participating institution. Three interviews were not conducted because one leader had COVID-19, one leader did not respond, and one leader’s child had an emergency.

Considering the planned group interviews, one with social workers was not conducted due to challenges during a pandemic, but one with frontline obstetric staff was possible. Leaders invited the participants, who were then self-selected. Six healthcare workers from four participating maternity hospitals—physicians, nurses, and technical nurses—formed these groups.

#### Individual interviews

Interview analyses were categorised as follows:

- **Category 1**. The equity and anti-racism training contributed to a more profound recognition of race and racism awareness, leading to a change in culture.
- **Category 2**. Priority change ideas and actions focusing on anti-racism and equity were demonstrated in several ways among the leaders.
- **Category 3**. Challenges when equity is centralised in the care model.

Overall, the recognition of racism evolved among leaders and institutions. Following the training, all leaders identified racism as a priority. The training fostered cultural change within their teams, aiding their understanding of how structural racism plays a crucial role in patient care throughout Brazilian history. The leaders viewed this anti-racist journey as personal but also recognized the need for further interpersonal and institutional commitments and actions to tackle inequities and racism in the system.

##### Category 1

Most leaders interviewed reported a noticeable cultural change in their maternal care units. Recognising structural racism and the privileges stemming from this model allowed participants to concentrate on creating spaces for discussion and institutional adaptations, promoting teamwork and enhancing patient care. Moreover, understanding how socioeconomic status, class, and race risks are interrelated shows insight into how intersectionality also affects women’s outcomes. Table 4 presents the main quotes from the individual leaders’ interviews in this category.

**Table 4:** Main quotes from the individual interviews– Categories 1 to 3

Category	Topic	Most representative quotes
Category 1	Impact of equity and anti-racist training	“*It made us have a better understanding of our processes. Through the cases presented*,* we learn and can look at the process concerning how we interact with women assisted…We also adapted to improve this system*” **Interview 3**
	Understanding racism helped leaders to prioritize the creation of spaces to confront racism.	*“It is not seen as something real and visible*,* but it is present in interpersonal relationships*,* whether between physician and patient*,* nurse and technician*,* or technician and patient… Racism is here*,* so we must make people see it to fight it. This is our goal: through conversation*,* to fight structural racism.”* **Interview 13**
	Internal analysis of the cultural transformation process as well as the discussion for prioritizing and translating knowledge into action	*“It really made us change ourselves and each person we talk to. It was also shown the importance of passing this to someone*,* talking to people*,* and exposing all of this. So*,* I believe it has completely transformed us; it is a seed that was planted.”* **Interview 4**
	Tensions arise when leaders act and prioritize anti-racism in their care settings.	*“I have been prioritizing this long since I joined*,* but the physicians do not pay attention [to women at socioeconomic risk]. People do not pay much attention to these mothers… Sometimes they ignore them. There is a problem of not wanting to deal with the patient*,* which is a problem for everyone in the hospital team.”* **Interview 17**
	Leaders and hesitation about racism being real	*“We have a lot of mixed and black people here. We do not see much [Racism] here and do not witness it*,* at least in our environment. We have no reports of racism.”* ***Interview 16***
Category 2	Use of data stratified by race and ethnicity for analysis	*“We now stratify all indicators based on race. The team received training to approach the patient about the colour with which he/she identifies.”* **Interview 11**
	Institutions analyzed their indicators and stratified data to understand and act on inequities in the system.	*“Right now*,* we have tools to do that. We have tools to access data by race: anaesthesia by race*,* complications by race…Today we also implemented a hospital information system. We filled out a form for near-miss events. This way*,* we also have a tool to evaluate near-miss events according to race… We created a small space here to create several indicators and work on this issue.”* **Interview 6**
	Dissemination of awareness and socialization of anti-racist education and socioeconomic risks within teams	*“We start with monthly or weekly meetings to discuss this issue. And then*,* we started to address the issue in triage*,* at the nursing station*,* with the doctors.”* **Interview 14**
	Transformation in a team that considers socioeconomic risk as part of women’s quality care at the intersection of poverty, racism, and other social health determinants	*“Understanding the vulnerability of these mothers was not seen before as important. So we started to collect and look for vulnerability —the ability to begin to understand our service— and have the patience to observe how we care for patients. This way*,* you start to present actions and reduce this vulnerability in the service. So I think that is what has been changing.”* **Interview 4**
	Identifying and prioritizing women at socioeconomic risk is a way to adapt the service to those needing attention.	*“We identify the socioeconomic risk as soon as they arrive at the unit. In the pre-delivery period*,* all employees can identify whether they need more attention*,* as they may not have access to health services. So*,* they should be screened*,* as they may have yet to have full access to prenatal care. Internally*,* the team can see this as a priority*,* which would already be the nurse’s job.”* **Interview 11**
	Cultural adaptations according to communities that present socioeconomic risks or risks of facing racism	*“We reach many indigenous women and focus on respecting their culture. Therefore*,* doing our job and respecting the culture is essential to provide the best service.”* **Interview 7**
Category 3	Understand the importance of the process and use the data to develop a more solid analysis of what equity actions are	*“In terms of the data*,* we were able to ask about race and ethnicity and have this indicator of maternal mortality stratified. What is missing from our action plan is what to do with this data.* **Interview 16**
	Tension and resistance to anti-racism by leaders, specific physicians, and other frontline professionals	*“When we bring the data*,* we feel much resistance from the medical team. When we say that mortality is higher in black women and that they die more from preventable causes*,* it is like they do not pay attention.”* **Interview 5**
	Challenges including staff time and COVID-19 pandemic restrictions	*“There are many comments about the lack of staff and the mental exhaustion experienced when people feel overwhelmed because when we arrive with a new proposal*,* even when it is important*,* care comes first. Whoever brings new projects or ideas for improvements is not well seen*,* but rather seen as the person who brought more work and problems.”* **Interview 19**
	Emotional resources and how the equity program affected listening	*“I think that showed me that listening to what people say is important. Even when you speak*,* everything becomes evident*,* and you begin to understand that person’s perspective and when he understood. So*,* I think feedback is essential.”* **Interview 4**
	Challenges when providing feedback to teams who engage in racist actions towards patients or other healthcare professionals	*“We did not think about that [incorporating feedback into these actions] because it is a very delicate approach. I do not even know how to do it during care… Sometimes you hear reports of racism*,* but there is little intervention in communicating with others about your racist action.”* **Interview 13**

##### Category 2

Before CAM, race and ethnicity were identified by health workers’ declarations–not by self-declarations– and data was not stratified. The obstetric teams could address and train on this topic based on leaders’ responses. Some team leaders initiated this awareness-raising process through monthly or weekly in-unit discussions or huddles, while others adopted individual approaches. The action was ultimately established when the clinical staff recognised and understood the vulnerabilities. Designing a distinct flow to identify the most vulnerable using a multidisciplinary strategy was essential to translate the acquired knowledge into daily actions. Interestingly, based on community settings, care adaptations generated different ideas for change depending on the context. For instance, many Haitian immigrants were in one hospital, where this motherhood included translators as part of the multidisciplinary team. Another hospital worked with the indigenous community, creating strategies to ensure that mothers’ customs were respected during care.

##### Category 3

This category highlights the challenges faced by institutions regarding the need for more time to commit to equity, the resistance from leaders and clinical staff, COVID-19 pandemic restrictions, and the lack of expertise necessary to extend an equity-oriented model beyond their units. Emotional and communication resources were somewhat impacted by the training; consequently, much remains to be accomplished, especially in providing feedback and developing accountability processes for identifying individuals who have engaged in racist actions.

#### Group interviews

Interview analyses were categorised as follows:

- **Category A**. There was the dissemination of information and actions from the participating leaders in the equity and anti-racist training, which primarily focused on adapting care for mothers at risk and discussions about race and ethnicity.
- **Category B**. Challenges of getting more staff to talk about racism regarding limited resources and time, and priorities focused on clinical work.
- **Category C**. A healthcare professional dedicated explicitly to this equity work.

The actions guided by the CAM opened a discussion that was previously ignored. Even with this advance, it is recognised that this work is complementary to their clinical duties, being difficult to fit into the daily assistance. Interviews mentioned many times that their equity and anti-racism work was slow and could go faster if someone in the hospital was explicitly assigned to this type of work.

Based on group interview responses, the training evidently affected the leaders. They constantly conversed within teams and actively shared what they learned to facilitate their equity-oriented practices. All participants mentioned the importance of their leaders disclosing this information and inviting them to collaborative QI activities. Table 5 summarises the main quotes from these three categories.

**Table 5:** Main quotes from the group interviews– Category A to C

Topic	Most representative quotes
Dissemination of information by leaders to the team	*“The leader carries out actions. First*,* they are circles of conversation and action with awareness of the subject. Then*,* some important data were passed on to the need to implement changes in the institution. Individualized training was also carried out. That is when we started our journey*,* and*,* from then on*,* we were able to bring a focus group interview and other actions at this junction.”*
Strategies used by leaders to bring content and raise awareness about anti-racism	*“It is a dialogue to make professionals aware and establish a service process with a social perspective. All of this was specifically important to us. I managed to change my perspective about myself and the service.”*
Reflections on conversations about racism and the development of race consciousness among health professionals	*“Work is not just about pathology. In addition to social issues*,* I understand how racism in our daily lives manifests itself at work. We needed to focus on the issue and work with our team daily.”*
Actions by multidisciplinary teams that create a service model focused on equity.	*“Now we are discussing daily the scope of the equity project to treat these patients that we consider to be at higher risk*,* according to their own needs.”*
Challenges to developing an equity-oriented care model include staff time and the need for experience with equity.	*“It is essential to work with other teams to address equity*,* as no one is dedicated to this work*,* and each professional must work together. This work shows the importance of work equity. So we feel a small barrier in this work because there is no dedicated person. There is the question of the number of people we must bring to this equity work. We connect with other teams*,* professionals*,* psychologists*,* social workers*,* doctors*,* and nurses to develop a strategy.*
Challenges presented by interpersonal dynamics and physicians as mediators	*“The whole team is one of our challenges too*,* unfortunately*,* even the team leaders. They do not see each other*,* but there is a kind of racism*,* so we face many challenges. Awareness is critical in bringing these leaders into the conversation*,* and that is what we are always trying to do.”*

### Triangulation

#### Evidence of a better understanding of ethnic-racial inequalities, institutional racism, and racism recognition by the leaders and participating institutions

According to the quantitative method, most scores related to this topic exceeded 4.00, showing an improvement following the training. Additionally, based on qualitative methods, nearly all leaders felt that the training helped them identify racism and increased their understanding of ethnic-racial inequities in the system.

#### Resistance when trying to bring new content to the clinical staff, as well as a lack of tools when dealing with the emotional resources needed to confront interpersonal racism

Obstetric teams have been challenged by the lack of overt political processes for identifying and processing racism in interpersonal forms. In the quantitative component, the two lowest-ranking categories were prioritisation, motivation, and translation of knowledge into action. Interview responses referred to the difficulties of dealing with the resistance of health professionals who did not believe that racism was a social determinant of health. It was also often expressed that one of the biggest challenges was holding people accountable for perceived racist actions and the lack of time to deal with it.

## Discussion

Although national efforts, Brazil has faced difficulties in achieving a further reduction in pregnancy-related deaths [9–11]. Several factors can explain this epidemiological behaviour, with issues of equity and racism also potentially limiting better maternal outcomes, particularly in underrepresented populations [25–27].

System-wide inequities and racism are reflected in higher rates of morbidity and mortality among various women’s health indicators, including congenital syphilis, hypertensive disorders of pregnancy, HIV, COVID-19, and others, with poor and Black women being the most affected [7, 10, 11, 28–31]. There is a broad consensus in Afro-Brazilian research that awareness must be fostered to change cultural attitudes and prejudices in medical practices towards Black individuals or those from other at-risk ethnicities [17]. In this collaborative effort, the equity and anti-racism interventions aim to: (i) contribute to QI activities aimed at reducing iMMR, (ii) raise awareness about structural, institutional, and interpersonal racism within participating obstetrical teams, and (iii) understand how these issues manifest in the daily care of Brazilian mothers.

### Anti-racist education and training for healthcare professionals

Interventions in the Brazilian public healthcare system have been developed to enhance the recognition of inequalities and translate them into actions that provide more humane care. Most of these initiatives have occurred in smaller contexts, often focusing solely on equity and anti-racism in a few hospitals or units [32]. With the CAM, the scope extends to more institutions and healthcare professionals who directly assist vulnerable women; however, there is still much to achieve on both the national and international agendas [19, 33, 34].

An essential aspect is to deconstruct the perceptions of blackness that are regarded as the “usual” care, which is part of the institutionalised racism prevalent in Brazilian society. Without this type of intervention, as presented here, obstetric teams would continue to carry out daily inequities as if they were “normal” during medical assistance, and microaggressions would persist over time. The anti-racist training and equity learning sessions during the CAM emphasised these images and scenarios: what is normalised and socialised regarding blackness and poverty in the health care system. Grasping this issue is critical to achieving fair and ethical obstetric care [20, 35].

Part of the work carried out was to understand how the intersection of identities and the socioeconomic context plays a role in maternal outcomes [36]. Above all, it examined how the racism and gender biases exhibited by health workers pose risks for mothers throughout the system, not just in reproductive care [37]. For instance, Leal et al. [38]. explain that black women are often not believed when they request medication for pain during labour. Indeed, healthcare users perceive their experiences of racism as including inadequate care, dismissive attitudes toward their symptoms and suffering, a lack of respect, and diminished power to negotiate during healthcare interactions [39, 40]. These encounters with racism lead to a loss of trust in healthcare, increased unmet needs, and delays in seeking medical assistance [41, 42]. During the CAM, several leaders began to stratify process-level data, which allowed them to recognize these inequities and acknowledge how obstetric teams were involved in these actions. Systematic reviews [43–45] and a scoping review of qualitative and quantitative studies [46] indicate that healthcare staff unknowingly perpetuate racism through implicit racial bias. Particularly impactful for the leaders of the CAM was the observation of how institutionalized and structural systemic racism was deconstructed, highlighting how privileges and power have been shaped by generations of oppressive practices [21]. They also saw the importance of a cultural transformation to ensure that minorities do not receive deprioritized diagnoses and treatment [36, 47].

Although only leaders participated in the training, the entire team participated in the broader equity sessions, facilitating the development of a patient-centred care model in their respective institutions. These efforts included collaborating with a multidisciplinary team to equally prioritise socioeconomic and clinical risks and the bundles implemented during the CAM. Furthermore, most leaders initiated discussions– individually or in groups–in their maternal care units, demonstrating increased emotional and communication resources and enhancing feedback and support within the obstetric teams. Despite receiving adequate training, studies indicate that healthcare staff struggle to discuss racism in their workplace [48, 49]. More robust institutional frameworks on addressing racism are still necessary to effectively create the needed culture to combat racism in healthcare and adequately address the maternal mortality crisis [20, 35, 36, 47].

There was also a process of awareness and analysis through the understanding of their stratified data to identify inequities in care, which spurred a critical examination of what was occurring, prompting them to reflect on the factors generating these disparities in their processes. Although challenges were faced, this strategy for training leaders and social workers is recommended for the future [50]. Research indicates that leaders’ equity knowledge and an equity-oriented service model are essential for progress to achieve [50, 51]. One of the significant challenges encountered during CAM was the lack of time, resistance from some professionals, and not having a transparent partnership with the human resources department to formulate policies addressing instances of interpersonal racism.

One key lesson learned is the necessity of institutional commitment to long-term monitoring and reinforcement of anti-racist practices. Without ongoing assessments and structured follow-up mechanisms, it becomes challenging to measure the durability of knowledge retention and behavioural changes among healthcare professionals. Future interventions should focus not only on initial training but also on integrating periodic refresher courses, instituting policies for accountability, and continuously monitoring disparities in care delivery. Establishing a system that embeds equity-oriented practices within routine healthcare workflows will be critical for fostering sustainable change and ensuring that the progress made through quality improvement initiatives translates into lasting improvements in maternal health outcomes.

### Data as the mainstay of the equity-oriented model

The CAM utilized the four steps proposed by Barbara Loves to foster a liberating conscience as part of a process of re-socialization and anti-racist education [52]. Developing an equity-oriented service model fundamentally relies on understanding the system and its inequities through data. One of the prioritised actions–based on the DD and theory of change– was to integrate ethnicity into information systems and stratify the leading indicators based on iMMR within the maternal care unit. Although the *National Policy for Integral Health of the Black Population* provides a step-by-step guide for adapting care to Afro-Brazilian populations in hospital, municipal, and state management strategies in Brazil [17], most hospitals still do not stratify the data. Furthermore, they do not yet tailor their care to populations experiencing the most significant inequities in the system [19, 27, 53].

Data analysis from the Brazilian public health system has identified opportunities and emphasised the importance of training frontline and reception health professionals in correctly asking questions through self-identification. However, only a few have been able to institutionalise or maintain adequate data stratification [19, 54, 55]. Before the CAM, all leaders stated that the triage professional asked the ethnicity question, and women were not allowed to self-identify. This opportunity is crucial for identifying and tracking inequities in the care processes, reinforcing stratification as a powerful tool for a better understanding of equity-oriented obstetric care [27, 56, 57].

### Equity and anti-racism as a quality improvement approach

The CAM qualitative results underscore the necessity of employing a multidisciplinary team approach that includes individuals in administrative roles, leaders, racialised members, and community representatives [58]. All stakeholders–policymakers, healthcare systems, and healthcare providers– have a crucial role. This approach necessitates a solid application of improvement science and PDSA cycles to identify the most effective strategies for teams. Having a clear theory of change, understanding variations, and establishing clear objectives and targets for equity and anti-racism are all essential components for advancing and committing to the work and investing time and resources to achieve sustainable system-level change [58–60].

Equity and quality improvement are interconnected, and every project must have strict equity goals and ideas for change, as evidenced here. Future QI collaboratives should include data stratification on all key process indicators to ensure that leaders understand how inequities may affect their QI indicators.

### Limitations

Although our work demonstrates excellent results, it has limitations. First, one of the main issues is the selection criteria of the participating institutions. Based on the previous standards set by the “*Parto Adequado*” Collaborative, the selection of hospitals may not have been the most suitable for this initiative.

Second, the maternity hospitals come from various Brazilian states and may possess different levels of understanding and diverse dynamics concerning racism related to the populations they serve and their previous experiences. Additionally, institutional characteristics such as size, complexity, and management, among others, may complicate the external validation of our findings. Further studies are essential, including a broader population and subgroup analysis. Strengthening relationships with community-based organizations should also be considered when developing a change theory and initiating any large-scale collaboratives.

Third, while social workers offered critical insights into systemic inequities and institutional efforts toward anti-racism, the perspectives of healthcare workers providing bedside care were less explored. This was mainly due to logistical challenges, including the limited availability of clinical staff during the COVID-19 pandemic. Future studies should prioritise a more diverse range of interview participants to ensure balanced representation of perspectives from both social workers and healthcare professionals, thereby better capturing the multifaceted impact of equity-driven QI initiatives in maternal care. Additionally, because of the low number of leaders who responded to the pre-survey, conducting a full before-and-after analysis was not possible. Future collaborations should incorporate a stronger emphasis from the outset on restructuring teams and implementing more robust participatory management strategies. These strategies should address the challenges of an equity-oriented care model and aim to strengthen partnerships.

Fourth, there is an absence of long-term follow-up assessments to evaluate the retention and practical application of knowledge gained through anti-racism training. While the pre- and post-test surveys offered valuable insights into immediate changes in awareness and attitudes, they do not capture the sustainability of these effects over time. The lack of longitudinal monitoring prevents us from determining whether the observed improvements in knowledge and awareness translated into sustained behavioural change within clinical practice. Future research should include repeated follow-up assessments at multiple time points post-intervention to evaluate the long-term impact of anti-racism training on healthcare professionals and maternal health outcomes.

Finally, COVID-19 was a major issue in the last year of the project and should, therefore, have been considered when it was impossible to approach all the leaders or social workers who participated.

## Conclusion

To the best of our knowledge, the CAM is the first QI initiative in Brazil to include equity and anti-racism in the theory of change and clinical outcomes indicators as a priority rather than merely a complementary component. Maternal units began adapting to promote equity-oriented care, resulting in a positive cultural transformation in daily assistance. With a significant reduction in iMMR, the CAM demonstrates that a QI intervention addressing inequities and racism issues is a feasible and promising approach to improving maternal outcomes within an equity-oriented model of care.

## Electronic supplementary material

Below is the link to the electronic supplementary material.

Supplementary Material 1

## Data Availability

No datasets were generated or analysed during the current study.

## References

[1] World Health Organization. Maternal mortality: Levels and trends 2000–2020. Available at: https://www.who.int/publications/i/item/9789240068759 (accessed on March 8, 2025).

[2] Alkema L, Chou D, Hogan D, et al. Global, regional, and national levels and trends in maternal mortality between 1990 and 2015, with scenario-based projections to 2030: a systematic analysis by the UN maternal mortality estimation inter-agency group. Lancet. 2016;387:462. 26584737 10.1016/S0140-6736(15)00838-7 PMC5515236

[3] Kassebaum NJ, Bertozzi-Villa A, Coggeshall MS, et al. Global, regional, and national levels and causes of maternal mortality during 1990–2013: a systematic analysis for the global burden of disease study 2013. Lancet. 2014;384:980. 24797575 10.1016/S0140-6736(14)60696-6 PMC4255481

[4] World Health Organization. Standards for improving quality of maternal and newborn care in health facilities. Available at: https://www.who.int/publications/i/item/9789241511216 (accessed on March 8, 2025).

[5] Petersen EE, Davis NL, Goodman D, et al. Racial/ethnic disparities in pregnancy-related deaths - United States, 2007–2016. Morb Mortal Wkly Rep. 2019;68:762. 10.15585/mmwr.mm6835a3 PMC6730892 31487273

[6] Wang E, Glazer KB, Howell EA, et al. Social determinants of pregnancy-related mortality and morbidity in the United States: a systematic review. Obstet Gynecol. 2020;135:896. 32168209 10.1097/AOG.0000000000003762 PMC7104722

[7] Empresa Brasil de Comunicação. Agência Brasil. Available at: https://agenciabrasil.ebc.com.br/saude/noticia/2018-05/maioria-de-mortes-maternas-no-pais-ocorre-entre-mulhere-negras-jovens (accessed on March 8, 2025).

[8] Secretaria de Vigilância em Saúde. Painel de monitoramento da mortalidade materna. 2022. Available at: http://svs.aids.gov.br/dantps/centrais-de-conteudos/paineis-de-monitoramento/mortalidade/materna (accessed on March 8, 2025).

[9] Leal LF, Malta DC, Souza MFM, et al. Maternal mortality in Brazil, 1990 to 2019: a systematic analysis of the global burden of disease study 2019. Rev Soc Bras Med Trop. 2022;55:e0279. 35107531 10.1590/0037-8682-0279-2021 PMC9009438

[10] Tenorio DS, Brasil AGM, Nogueira BG, et al. High maternal mortality rates in Brazil: inequalities and the struggle for justice. Lancet Reg Health Am. 2022;14:100343. 35935590 10.1016/j.lana.2022.100343 PMC9346430

[11] Orellana J, Jacques N, Leventhal DGP, et al. Excess maternal mortality in Brazil: regional inequalities and trajectories during the COVID-19 epidemic. PLoS ONE. 2022;17:e0275333. 36264994 10.1371/journal.pone.0275333 PMC9584504

[12] Morton CH, VanOtterloo LR, Seacrist MJ, et al. Translating maternal mortality review into quality improvement opportunities in response to pregnancy-related deaths in California. J Obstet Gynecol Neonatal Nurs. 2019;48:252–62. 30981725 10.1016/j.jogn.2019.03.003

[13] Waiswa P, Manzi F, Mbaruku G, et al. Effects of the EQUIP quasi-experimental study testing a collaborative quality improvement approach for maternal and newborn health care in Tanzania and Uganda. Implement Sci. 2017;12:89. 28720114 10.1186/s13012-017-0604-x PMC5516352

[14] Hagaman AK, Singh K, Abate M, et al. The impacts of quality improvement on maternal and newborn health: preliminary findings from a health system integrated intervention in four Ethiopian regions. BMC Health Serv Res. 2020;20:522. 32513236 10.1186/s12913-020-05391-3 PMC7282234

[15] Howell EA, Brown H, Brumley J, et al. Reduction of peripartum racial and ethnic disparities: a conceptual framework and maternal safety consensus bundle. Obstet Gynecol. 2018;131:770. 29683895 10.1097/AOG.0000000000002475

[16] Borem P, Gushken AKF, Gushken AP, et al. A comprehensive strategy to mitigate institutional maternal mortality: lessons from a quality improvement initiative in Brazilian maternity hospitals. Glob Health Sci Pract (accepted on January 20, 2025). 10.9745/GHSP-D-24-00130 PMC12838806 41494826

[17] Ministério da Saúde, Secretaria de Gestão Estratégica e Participativa, Departamento de Apoio à Gestão Participativa e ao Controle Social. Política Nacional de Saúde Integral da População Negra, Uma Política do SUS. 2017. Available at: https://bvsms.saude.gov.br/bvs/publicacoes/politica_nacional_saude_populacao_negra_3d.pdf (accessed on March 8, 2025).

[18] De Araujo EM, Costa MCN, Hogan VK, et al. A utilização Da variável Raça/cor Em Saúde pública: possibilidades e limites. Interface (Botucatu). 2009;13:31.

[19] Chehuen Neto JA, Fonseca GM, Brum IV, et al. Política Nacional de Saúde integral Da população Negra: Implementação, conhecimento e aspectos socioeconômicos Sob a perspectiva Desse Segmento populacional. Saúde Coletiva. 2015;20:6. 10.1590/1413-81232015206.17212014 26060969

[20] Silva NN, Favacho VBC, Boska GA, et al. Access of the black population to health services: integrative review. Rev Bras Enferm. 2020;73:4. 10.1590/0034-7167-2018-0834 32490989

[21] Okun T. White Supremacy Culture– Still Here. 2021. Available at: http://www.whitesupremacyculture.info/uploads/4/3/5/7/43579015/white_supremacy_culture_-_still_here.pdf (accessed on March 8, 2025).

[22] The Institute for Healthcare Improvement. The breakthrough series: IHI’s collaborative model for achieving breakthrough improvement: Institute for Healthcare Improvement. 2003. Available at: http://www.ihi.org/resources/Pages/IHIWhitePapers/TheBreakthroughSeriesIHIsCollaborativeModelforAchievingBreakthroughImprovement.aspx (accessed on March 8, 2025).

[23] Ogrinc G, Davies L, Goodman D, et al. SQUIRE 2.0 (Standards for quality improvement reporting excellence): revised publication guidelines from a detailed consensus process. BMJ Qual Saf. 2016;25:986–92. 26369893 10.1136/bmjqs-2015-004411 PMC5256233

[24] Malawa Z, Spellen S, Gaarde J. A series of tools to advance Racial equity in the workplace. In *Race equity 101*. San Francisco, CA. Expecting Justice; 2019.

[25] Davis DA. Obstetric racism: the racial politics of pregnancy, labor, and birthing. Med Anthropol. 2018;37:1–14. 30521376 10.1080/01459740.2018.1549389

[26] Liu SY, Fiorentini C, Bailey Z, et al. Structural racism and severe maternal morbidity in New York State. Clin Med Insights: Women’s Health. 2019.;12:1179562X19854778. 10.1177/1179562X19854778 PMC8842459 35237092

[27] Hardeman RR, Kheyfets A, Mantha AB, et al. Developing tools to report racism in maternal health for the CDC maternal mortality review information application (MMRIA): findings from the MMRIA racism & discrimination working group. Matern Child Health J. 2022;26:661–9. 34982327 10.1007/s10995-021-03284-3

[28] Barbosa MS, Lima LA, Ribeiro SM, et al. Epidemiological study in Brazilian women highlights that syphilis remains a public health problem. Rev Inst Med Trop Sao Paulo. 2021;63:e4. 33533807 10.1590/S1678-9946202163004 PMC7845940

[29] Cunha AP, Cruz MM, Pedroso M. Análise da tendência da mortalidade por HIV/AIDS segundo características sociodemográficas no Brasil, 2000 a 2018. Ciência & Saúde Coletiva. 2022;27:895–908. 10.1590/1413-81232022273.00432021 35293467

[30] Lewis-Evans K, Day-Page L. Selected women’s health disparities. US Pharm. 2022;47:17–21.

[31] MacDorman MF, Thoma M, Declcerq E, et al. Racial and ethnic disparities in maternal mortality in the United States using enhanced vital records, 2016–2017. Am J Public Health. 2021;111:1673–81. 34383557 10.2105/AJPH.2021.306375 PMC8563010

[32] Batista LE, Rattner D, Kalckmann S, et al. Humanização Na Atenção à Saúde e as desigualdades raciais: Uma proposta de intervenção. Saude Soc. 2016;25:689–702.

[33] Amin A, Remme M, Allotey P, et al. Gender equality by 2045: reimagining a healthier future for women and girls. BMJ. 2021;373:n1621. 34183331 10.1136/bmj.n1621 PMC8237157

[34] Bates LM, Hankivsky O, Springer KW. Gender and health inequities: a comment on the final report of the WHO commission on the social determinants of health. Soc Sci Med. 2009;69:1002–4. 19665829 10.1016/j.socscimed.2009.07.021

[35] Eurico MC. A Percepção do Assistente social Acerca do Racismo institucional. Serv Soc Soc. 2013;114:290–310.

[36] Crear-Perry J, Correa-de-Araujo R, Johnson TL, et al. Social and structural determinants of health inequities in maternal health. J Womens Health (Larchmt). 2021;30:230–5. 33181043 10.1089/jwh.2020.8882 PMC8020519

[37] Patterson EJ, Becker A, Baluran DA. Gendered racism on the body: an intersectional approach to maternal mortality in the United States. Popul Res Policy Rev. 2022;41:1261–94.

[38] Leal MC, Gama SGN, Pereira APE et al. A Cor Da Dor: iniquidades raciais Na Atenção pré-natal e Ao parto no Brasil. Cadernos De Saúde Pública, 33(Cad. Saúde Pública, 2017 33 suppl 1), e00078816. 10.1590/0102-311X00078816 28746555

[39] Sim W, Lim WH, Ng CH, et al. The perspectives of health professionals and patients on racism in healthcare: a qualitative systematic review. PLoS ONE. 2021;16:e0255936. 34464395 10.1371/journal.pone.0255936 PMC8407537

[40] Peek ME, Odoms-Young A, Quinn MT, et al. Racism in healthcare: its relationship to shared decisionmaking and health disparities: a response to Bradby. Soc Sci Med. 2010;71:13–7. 20403654 10.1016/j.socscimed.2010.03.018 PMC3244674

[41] Harris RB, Cormack DM, Stanley J. Experience of racism and associations with unmet need and healthcare satisfaction: the 2011/12 adult New Zealand health survey. Aust N Z J Public Health. 2019;43:75–80. 30296819 10.1111/1753-6405.12835

[42] Powell W, Richmond J, Mohottige D, et al. Medical mistrust, racism, and delays in preventive health screening among African-American men. Behav Med Wash DC. 2019;45:102–17. 10.1080/08964289.2019.1585327 PMC8620213 31343960

[43] Paradies Y, Truong M, Priest N. A systematic review of the extent and measurement of healthcare provider racism. J Gen Intern Med. 2014;29:364–87. 24002624 10.1007/s11606-013-2583-1 PMC3912280

[44] Hall WJ, Chapman MV, Lee KM, et al. Implicit racial/ethnic bias among health care professionals and its influence on health care outcomes: a systematic review. Am J Public Health. 2015;105:e60–76. 26469668 10.2105/AJPH.2015.302903 PMC4638275

[45] FitzGerald C, Hurst S. Implicit bias in healthcare professionals: a systematic review. BMC Med Ethics. 2017;1:19. 10.1186/s12910-017-0179-8 PMC5333436 28249596

[46] Maina IW, Belton TD, Ginzberg S, Singh A, Johnson TJ. A decade of studying implicit racial/ethnic bias in healthcare providers using the implicit association test. Soc Sci Med. 1982;2018:219–29. 10.1016/j.socscimed.2017.05.009 28532892

[47] Silva AM. Gostando mais de nós mesmos: perguntas e respostas sobre auto-estima e questão racial. AMMA-Psique e Negritude (Ed.),2^a^ Edição ampliada, São Paulo, SP. Editora Gente; 1999.

[48] Holland AE. The lived experience of teaching about race in cultural nursing education. J Transcult Nurs. 2015;26:92–100. 24682320 10.1177/1043659614523995

[49] Sotto-Santiago S, Mac J, Duncan F, et al. "I didn’t know what to say": responding to racism, discrimination, and microaggressions with the OWTFD approach. MedEdPORTAL. 2019;16:10971. 10.15766/mep_2374-8265.10971 PMC7394349 32754635

[50] Hamed S, Bradby H, Ahlberg BM, et al. Racism in healthcare: a scoping review. BMC Public Health. 2022;22:988. 35578322 10.1186/s12889-022-13122-y PMC9112453

[51] Suarez K. The Role of Senior Leaders in Building a Race Equity Culture. 2018. Available in: https://www.bridgespan.org/insights/library/organizational-effectiveness/senior-leaders-role-in-building-race-equity/ (accessed on March 8, 2025).

[52] Love BJ. Developing a liberatory consciousness. In M. Adams (Ed.), *Readings for diversity and social justice*. New York, NY. Routledge;2000.

[53] Santos JE, Santos GCS. Narrativas Dos profissionais Da Atenção primária sobre a política Nacional de Saúde integral Da população Negra. Saúde Em Debate. 2013;37:563–70.

[54] Braz RM, Oliveira PTR, Reis AT, et al. Avaliação Da completude Da variável Raça/cor Nos sistemas nacionais de Informação Em Saúde Para Aferição Da equidade étnico-racial Em indicadores Usados Pelo índice de desempenho do sistema Único de Saúde. Saúde Debate. 2013;37:554–62.

[55] Batista LE, Monteiro RB, Medeiros RA. Iniquidades raciais e Saúde: O Ciclo Da política de Saúde Da população Negra. Saúde Em Debate. 2013;37:681–90.

[56] Dayo E, Christy K, Habte R. Health in colour: black women, racism, and maternal health. Lancet Reg Health Am. 2023;17:100408. 36531129 10.1016/j.lana.2022.100408 PMC9735204

[57] Krieger N. Discrimination and its consequences for health. Int J Health Serv. 2014;44:643–710. 25626224 10.2190/HS.44.4.b

[58] Hassen N, Lofters A, Michael S, et al. Implementing anti-racism interventions in healthcare settings: a scoping review. Int J Environ Res Public Health. 2021;18:2993. 33803942 10.3390/ijerph18062993 PMC8000324

[59] Taylor JK. Structural racism and maternal health among black women. J Law Med Ethics. 2020;48:506–17. 33021163 10.1177/1073110520958875

[60] Yearby R, Clark B, Figueroa JF. Structural racism in historical and modern US health care policy. Health Aff (Millwood). 2022;41:187–94. 35130059 10.1377/hlthaff.2021.01466

