# Supplementary material for: Strengthening equity and anti-racism in women’s care: a quality improvement initiative reducing institutional maternal mortality in Brazil
Source: Int J Equity Health. 2025 Apr 23;24:111. doi: 10.1186/s12939-025-02452-z (PMC12016309; doi:10.1186/s12939-025-02452-z)
Supplement: Supplementary file 1 — Supplementary Material 1 [file 12939_2025_2452_MOESM1_ESM.docx]

**SUPPLEMENTARY MATERIAL**

**Supplementary Table 1S**. Logical model of the (*blind*)


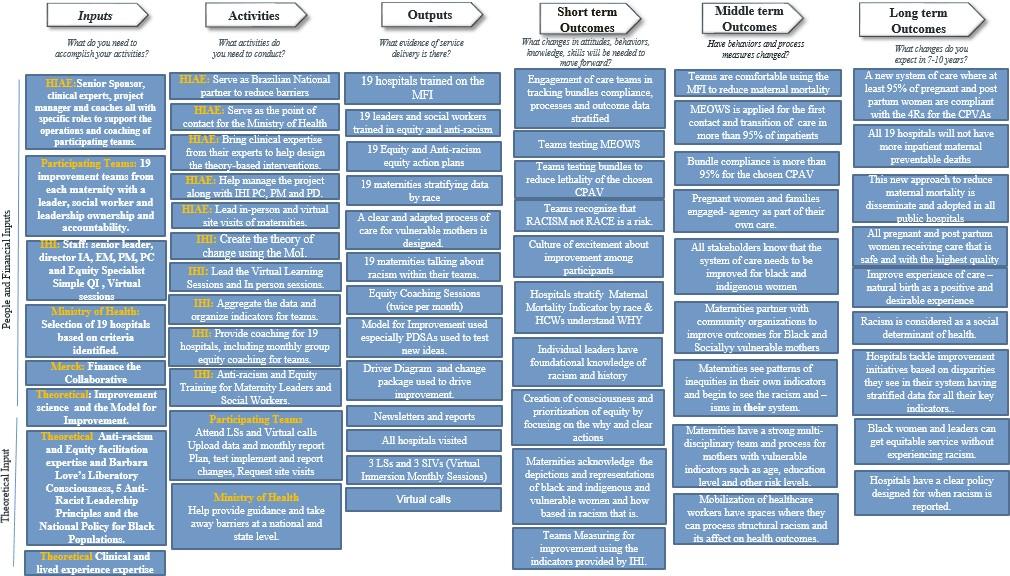


**Supplementary Table 2S**. The *Anti-Racist Leadership Survey*

| **Domains** | **Item** | **Statement** |
| --- | --- | --- |
| **Fundamental knowledge** | 1 | I understand the importance of gathering questions about race and ethnicity. |
|  | 2 | I understand the Brazilian legacy against black and mixed-race culture and its link with the inequitable results from all institutions nowadays. |
|  | 3 | I am aware of my organization's anti-racist priorities and my role in these initiatives. |
|  | 4 | I can recognize a microaggression if it occurs. |
|  | 5 | I can confidently and proudly report employee demographics (current and future trends) for my team and organization. |
|  | 6 | I know what to do when a racist act occurs at work. |
|  | 7 | I can observe how racism manifests itself institutionally, socially and structurally. |
|  | 8 | I understand the difference between fairness and equality. |
| **Race and Racism awareness** | 1 | I actively and critically evaluate factors influencing my values, beliefs, and preferences and discuss my views with others. |
|  | 2 | I try to acknowledge my biases and seek different perspectives before making decisions and/or contributing to people's decisions (e.g., recruiting, rewarding, evaluating, and developing). |
|  | 3 | Demonstrate knowledge and awareness of the historical, social, environmental, emotional, mental and economic issues of racism and its impacts on the local communities we serve, our employees and our work. |
|  | 4 | I like, value and treat with respect people from different backgrounds and levels in the organization. Based on our positive, meaningful, consistent interactions, I can also say this. |
|  | 5 | I am aware of the privilege I have and leverage it visibly and subtly to include people and discourage exclusion. |
|  | 6 | I can identify racism as soon as it occurs. |
|  | 7 | I have a racially diverse group of friends at work. |
|  | 8 | I can observe how racism has different impacts on different colour groups. |
| **Motivation and prioritization** | 1 | In meetings, I demonstrate bold leadership, consistent with a racial equity perspective and an understanding of power and privilege. |
|  | 2 | I create a work environment where initiatives to establish an anti-racist culture can be openly discussed and defended. |
|  | 3 | I constantly educate myself and others about racism. |
|  | 4 | I frequently ask colleagues (including collaborators) for feedback on the impact of what I say and do in informal conversations and/or proper feedback tools. |
|  | 5 | I proactively seek opportunities to mentor, train and encourage others from underrepresented racial groups. |
|  | 6 | I discuss the benefits of an anti-racist, diverse and inclusive organization with colleagues based on professional and personal experiences. |
|  | 7 | I work to create an environment where everyone feels valued. |
|  | 8 | I recognize that behaviours and words can have different meanings in different cultures, and I try to learn about cultures other than my dominant culture by establishing authentic and meaningful relationships with people from diverse racial, ethnic, and educational backgrounds, as well as different sexual orientations, ages, and genres. |
| **Translation of the knowledge in action** | 1 | Internal and external patients' demographics (current and future trends) define where and how I recruit and select people and/or advise others about recruiting and selection. |
|  | 2 | Internal and external contributors (current and future trends) define where and how I recruit and select people and/or advise others about recruitment and selection. |
|  | 3 | I analyze data and solicit multiple perspectives to ensure that the outcomes of employees' decisions and/or those I contribute to are racially equitable, fair, and consistent. |
|  | 4 | When developing and/or implementing my plans, I consider racial impact as part of my plan for my team and our target community. |
|  | 5 | I integrate racial justice knowledge, skills, and practices into performance objectives (such as job descriptions and work plans) and staff considerations/evaluations. |
|  | 6 | I can provide appropriate racially/racial equity services to employees and the local communities we serve. |
|  | 7 | I actively strategize and work in partnership with others to advance anti-racist work. |
| **Emotional resources and communication** | 1 | I say what I am thinking and feeling, taking other people into account and equating my actions with my words and values. |
|  | 2 | I provide constructive feedback to everyone I manage and/or teach/assist and extract their perspectives on situations while actively listening and seeking understanding. |
|  | 3 | In interactions, I try to recognize and work closely with my racial biases, paying attention to the speaker, listening carefully without interrupting, observing verbal and nonverbal stimuli, and often checking for understanding. |
|  | 4 | I say or do something, visibly, practically or verbally, to disapprove when others stereotype, discriminate or treat people poorly based on their race and ethnicity. |
|  | 5 | I can manage the tensions and emotions that often arise regarding racism, diversity, equity, justice and inclusion. |
|  | 6 | To aid understanding, I objectively and frankly communicate my values, priorities, and needs regarding racial equity. |
|  | 7 | Daily, I warmly receive and act on feedback from people from different backgrounds and/or with different perspectives/experiences. |
|  | 8 | I have no problem bringing up issues of race and racism in meetings with my colleagues, supervisees/supervises. |
|  | 9 | I welcome feedback on my words or actions that may have been racist. |
|  | 10 | I have the competence to warn about racism. |
| **Total** | **42** |  |
